# Penicillamine ameliorates intestinal barrier damage in dextran sulfate sodium-induced experimental colitis mice by inhibiting cuproptosis

**DOI:** 10.3389/fimmu.2025.1580963

**Published:** 2025-09-03

**Authors:** Jiaze Ma, Yimeng Ma, Shuangshuang Wang, Wenwen Tang, Chen Chen, Yang Li, Yugen Chen, Tuo Chen

**Affiliations:** ^1^ Department of Colorectal Surgery, The Affiliated Hospital of Nanjing University of Chinese Medicine, Affiliated Hospital of Yangzhou University, Yangzhou, Jiangsu, China; ^2^ International Institute for Translational Chinese Medicine, School of Pharmaceutical Sciences, Guangzhou University of Chinese Medicine, Guangzhou, China; ^3^ Department of Endoscopy Center, Jiangsu Province Hospital of Chinese Medicine, Nanjing, China

**Keywords:** ulcerative colitis, inflammatory bowel disease, penicillamine, cuproptosis, intestinal barrier

## Abstract

**Background:**

Cuproptosis is a copper-dependent form of cell death. However, its role in ulcerative colitis (UC) remains unknown.

**Aim:**

To investigate whether cuproptosis is involved in UC and whether penicillamine (PA) improves colitis in mice by inhibiting cuproptosis.

**Methods:**

We analyzed the expression of cuproptosis-related genes in patients with UC using the Gene Expression Omnibus database. We used dextran sulfate sodium (DSS) to establish an experimental model of UC and explore the effects of cuproptosis on the intestinal barrier. Mice were treated with the copper-depleting agent tetrathiomolybdate to establish causality between cuproptosis and intestinal barrier damage in mice with DSS-induced colitis. We assessed the effects of PA on the intestinal barrier in mice with DSS-induced colitis. Key methodologies included copper quantification using inductively coupled plasma mass spectrometry and rubeanic acid histochemical staining, along with the analysis of cuproptosis-related and barrier proteins using qRT-PCR, immunoblotting, immunohistochemistry, and immunofluorescence.

**Results:**

Cuproptosis was closely related to intestinal barrier damage in patients with UC and in DSS-induced colitis mice, characterized by increased copper levels and dihydrolipoamide S-acetyltransferase (DLAT) oligomerization and reduced Fe-S cluster-containing proteins ferredoxin 1 (FDX1) and lipoyl synthase (LIAS) levels. Copper depletion ameliorated disease-related manifestations in mice with colitis, mitigated the aberrant expression of pro-inflammatory factors, and concurrently enhanced the expression of tight junction proteins. PA inhibited cuproptosis in the intestinal barrier of mice with colitis by reducing excess copper levels and DLAT oligomerization, as well as rescuing the loss of FDX1 and LIAS.

**Conclusion:**

Cuproptosis is involved in UC pathogenesis. The identification of PA, which inhibits cuproptosis in the intestinal barrier of mice with colitis, provides a novel therapeutic option for the clinical management of UC.

## 1 Introduction

Ulcerative colitis (UC), a chronic inflammatory bowel disease, is characterized by recurrent episodes of diarrhea, rectal bleeding, and abdominal pain, which severely reduce the quality of life of patients who experience severe symptoms. With the current global prevalence of UC estimated at 5 million cases and its increasing incidence worldwide, UC has become a global health challenge (1). UC typically occurs in individuals with a genetic susceptibility to environmental exposure and is closely related to intestinal epithelial barrier defects, microbiota dysbiosis, and immune response disorders (2–6). However, its complex pathogenesis of UC remains unclear. Therefore, delving further into the precise etiology and pathogenesis of UC in the aforementioned aspects has the potential to enhance its diagnosis, treatment, and prognosis.

The initial phase of UC pathogenesis is characterized by the disruption of the intestinal barrier and a loss of mucosal homoeostasis (7). This phase is closely associated with various types of cell death in the intestinal barrier, and regulation of the intestinal barrier is an important strategy for UC treatment (8–10). The mucosal layer and tight junctions of the intestinal barrier maintain the full functioning of the barrier in a healthy state by separating the microbiota of the intestine from the immune system of the mucosal surface. Tight junction protein expression may be altered as UC progresses, thereby impairing barrier function (11). In UC, intestinal barrier injury leads to persistent microbial colonization of the lumen and uncontrolled inflammatory responses (12). In addition to conventional regulation of cell death, UC is closely associated with specific metal ion-dependent cell death processes such as ferroptosis (13). Copper is a trace metal vital for many physiological functions in living organisms and is necessary for maintaining regular biological activities (14, 15). Excess copper kills cells, while insufficient copper prevents the enzymes responsible for copper binding from functioning (16). However, the effect of copper on the intestinal barrier in patients with UC remains unclear.

Cuproptosis is a recently discovered form of regulated cell death that depends on the accumulation of excess copper. It differs from all other known regulated cell death pathways, including apoptosis, necroptosis, pyroptosis, and ferroptosis (17). The reduction of proteins bearing the Fe-S cluster and lipoylated protein oligomerization are the mechanisms underlying this type of copper-dependent cell death. Lipoylation occurs in essential mitochondrial proteins, especially those within the pyruvate dehydrogenase complex involved in the tricarboxylic acid (TCA) cycle (18, 19). Following this modification, the lipoyl moiety acts as a direct copper-binding site. This binding leads to the oligomerization of the lipoylated proteins. The outcome is a surge in acute proteotoxic stress, resulting in cell death (17). Most patients with UC exhibit abnormal copper metabolism (20–22). Recent studies have reported an association between UC and cuproptosis through database analyses, and genes related to cuproptosis may contribute to the early UC diagnosis (23). Therefore, exploring the mechanism underlying cuproptosis in the intestinal barrier of patients with UC is important for the development of innovative therapeutic agents.

Our study is the first to report that copper accumulation-triggered cuproptosis contributes to disruptions in intestinal barrier integrity in UC, which is mediated by the oligomerization of dihydrolipoamide S-acetyltransferase (DLAT) and depletion of Fe-S cluster-containing proteins, including ferredoxin 1 (FDX1) and lipoyl synthase (LIAS). This study aims to provide a significant advancement beyond previous bioinformatics predictions of cuproptosis-UC associations. In addition, we observed that penicillamine (PA), a clinical heavy metal antidote, could improve intestinal inflammation and barrier function by inhibiting cuproptosis in the colonic mucosa, thereby ameliorating UC. These findings provide preclinical evidence for expanding the indications of PA and provide a novel avenue for UC treatment.

## 2 Materials and methods

### 2.1 Human colonic tissue samples

Colon mucosa specimens were obtained from healthy controls and symptomatic patients with UC (Mayo endoscopic score ≥ 2) (24) at the Jiangsu Hospital of Traditional Chinese Medicine. Human colonic tissue samples comprised colon mucosa specimens obtained via colonoscopy biopsies. The exclusion criteria applied were patients in clinical or endoscopic remission (defined as Mayo endoscopic score ≤ 1); those with severe complications, including colorectal cancer, toxic megacolon, or intestinal perforation; and those with other chronic gastrointestinal diseases, such as Crohn’s disease or infectious colitis. All participants were adults aged 18–65 years old. Demographic characteristics are provided in **Supplementary Material S2**. Informed consent was obtained from all the study participants. The procurement of these specimens was approved by the Ethics Committee of Jiangsu Hospital of Traditional Chinese Medicine (permission no. 2022NL-083-01).

### 2.2 Analysis of differential expression and microarray data

The GPL20115 system was used for the microarray analysis of the gene expression datasets GSE66407, GSE75214, and GSE87466 from the Gene Expression Omnibus (GEO) collection. Variation in expression analysis was performed using the “Limma” program in R software. The following criteria were used to classify differentially expressed genes: (1) |log2 (fold change)| > 1 and (2) adjusted P < 0.05. Differential expression of cuproptosis-related genes was analyzed between patients with UC and healthy controls. Visualizations, including a heatmap, box plot, and Venn diagram, were created using the R packages “pheatmap,” “ggplot2,” and “venneuler,” respectively.

### 2.3 Animal experiments

Male C57BL/6 mice (6–8 weeks old) were used in each batch of experiments. Mice were housed in environmentally controlled rooms with a fixed temperature range, 50% humidity, and a 12-h light-dark cycle. Pilot studies confirmed the efficacy of tetrathiomolybdate (TTM)/penicillamine (PA) monotherapy in healthy mice (**Supplementary Figure S3**). Therefore, formal experiments focused on the efficacy in the disease context. TTM, a potent copper chelator, was used to validate the role of cuproptosis in colitis. The clinically used copper-chelating drug PA was evaluated for its therapeutic potential against DSS-induced colitis. To investigate the association between cuproptosis and colitis, mice were randomly assigned to three groups: control, dextran sulfate sodium (DSS), and TTM (323446; Sigma-Aldrich, Darmstadt, Germany, 0.7 mg/mice/day) with DSS after acclimatization for 7 days. Experimental colitis was induced by administering 2.5% (w/v) DSS (0216011090; MP Biomedicals, Santa Ana, USA) in drinking water for 7 days as previously reported (25). The mice in the control group drank water simultaneously. The dosing for TTM was based on previous research findings (26), while the corresponding DSS mice were gavaged with drinking water. To confirm the effects of PA on colitis and identify the optimal therapeutic dosage, mice were randomly divided into five groups: control, DSS, low dose PA (PA-L, 0.09 g/kg/day) with DSS, high dose PA (PA-H, 0.18 g/kg/day) (H31022286; Rare, Shanghai, China) (27) with DSS, and 5-aminosalicylic acid (5-ASA, 200 mg/kg/day) (A3537; Sigma-Aldrich) with DSS groups after acclimatization for 7 days. Simultaneously, mice in the PA or 5-ASA (positive control) groups were administered PA or 5-ASA via oral gavage once daily, whereas the control and untreated colitis mice received equal quantities of water. The disease activity index (DAI) (28), which encompasses factors such as body weight reduction, stool consistency, and rectal bleeding, was measured daily. The full-scoring rubric is provided in **Supplementary Material S1**. The Ethics Committee of the Affiliated Hospital of Nanjing University of Chinese Medicine approved all mouse tests and methods, which were carried out in compliance with the National Institutes of Healthcare Regulation for the Care and Utilization of Laboratory Animals (Approval No. 2024DW-006-01).

### 2.4 Histopathological analysis

For histopathological analysis, colon tissues were fixed in 4% paraformaldehyde (P6148; Sigma) for 24 h, embedded in paraffin, sectioned at a thickness of 5 μm, and stained with hematoxylin and eosin. The sections were scored microscopically by two pathologists blinded to the grouping information. Pathological assessments included the level of inflammatory cell invasion (0, none; 1, heightened in the lamina propria; 2, infiltration in the submucosa; 3, transmural infiltration) as well as the extent of tissue damage (0, no damage; 1, scattered lesions in the epithelium; 2, anabrosis or localized ulcers; 3, serious harm with widely used ulcers permeating the intestinal wall) (29).

### 2.5 Immunohistochemical and immunofluorescent staining

Colon slices were treated with 3% hydrogen peroxide to inhibit endogenous peroxidase activity before immunohistochemical staining. To identify the antigens, the pieces were heated in EDTA buffer (pH 8.0) for 3 min. The sections were blocked using 10% goat serum (G9023; Merck, Darmstadt, Germany) and incubated with the relevant primary antibody. Two experienced pathologists blinded to the group assignments scored the IHC sections, which were analyzed using ImageJ software.

After antigen retrieval, the sections were incubated with the antibody targeting the primary protein for 12 h at 4°C to perform double immunofluorescence staining. An additional biotin-conjugated antibody, along with streptavidin Alexa Fluor 488 or Cy3 (ab253268 or ab6939, respectively; Abcam), was used in succession. The slices were treated to identify secondary proteins after the initial staining was completed. 4′6-diamidino-2-phenylindole dihydrochloride (P36931, Invitrogen, Carlsbad, CA, USA) was used to stain nuclei. **Supplementary Material S1** contains a list of the antibodies used.

### 2.6 Immunoblot analysis

Colon tissues from the mice were sliced longitudinally and incubated in phosphate-buffered saline containing 10 mM EDTA for 1 h. Epithelial cells were lysed using radioimmunoprecipitation and sodium dodecyl sulfate sample buffers (P0015L; Beyotime, Shanghai, China). The protocol for isolation of murine colonic epithelial cells was performed according to a previously reported method (30). A bicinchoninic acid technique was used to assess the quantity of protein, and a bovine serum albumin reference curve was used to normalize the results. Following SDS-PAGE, proteins isolated from the colonic mucosa and epithelium were transferred onto nitrocellulose membranes. Membranes were blocked using 5% skim milk for 2 h at 26°C. The primary antibodies to FDX1 (1:1000), lipoic acid (1:1000), LIAS (1:1000), DLAT (1:2000), β-actin (1:20000), occludin (1:5000), and zonula occludens 1 (ZO-1; 1:5000) were diluted in western antibody diluent (P0023A; Beyotime) according to the manufacturer’s instructions. The membranes were then incubated with the primary antibodies for 12 h at 4°C, followed by the addition of the corresponding peroxidase-conjugated secondary antibody conjugates. ECL chemiluminescence was used to visualize the signals (BL520AB; BioSharp, Hefei, China). Western blots were subjected to densitometric analysis using NIH Image software. **Supplementary Material S1** contains a list of antibodies used.

### 2.7 Quantitative real-time PCR

Total RNA was isolated from colonic mucosal/epithelial tissues, and cDNA was produced according to the manufacturer’s instructions (Vazyme, Nanjing, China). An ABI 7500 real-time PCR apparatus (Applied Biosystems) and AceQ Universal SYBR qPCR Master Mix (Q711; Vazyme) were used for the qPCR. The results were normalized to β-actin levels. The cycling conditions and primer sequences for these genes are provided in **Supplementary Material S1**.

### 2.8 Rubeanic acid copper staining

Tissues were preserved in 10% neutral formalin (15512; Sigma-Aldrich), prepared for routine dehydration and embedding, and then sectioned at 5-μm thickness. Deparaffinization was performed using water. The experimental and positive-control slides were placed in purified water. The sections were subsequently stained with a rubeanic acid solution (1.00629; Merck) at 37°C for 16–48 h. After rinsing with 70% ethanol and a brief wash with distilled water, the slides were air-dried, and excess moisture was removed. Nuclear Fast Red staining was performed for 1 min, followed by rinsing with distilled water. Sections were routinely dehydrated to transparency and mounted on a neutral mounting medium.

### 2.9 Inductively coupled plasma-mass spectrometry

To measure total copper levels in the colonic mucosal tissue, the tissue was weighed and homogenized using non-metallic grinding beads. The resulting homogenates were digested with 65% nitric acid for 3 h (1.00443; Merck). After digestion, the volume of each sample was adjusted to 5 mL with ultrapure water. The samples were then examined using ICP-MS. The normalizing variable used in measuring total copper was tissue weight. Copper concentration standards in nitric acid were used to calibrate the standard curve for each experiment. Details of the ICP-MS conditions are provided in **Supplementary Material S1**.

### 2.10 Statistical analysis

Every outcome is shown as mean ± SEM. Two distinct categories were compared using an independent, unpaired, two-tailed t-test, and assessments between multiple groups were performed using one-way analysis of variance and Dunnett’s test. Statistical significance was defined as P < 0.05. Unless otherwise indicated in the figure legends, all experimental results were based on six mouse or human samples per group, and all experiments were repeated three times.

## 3 Result

### 3.1 Essential role of cuproptosis in the colonic mucosa of UC

**Figure 1:**
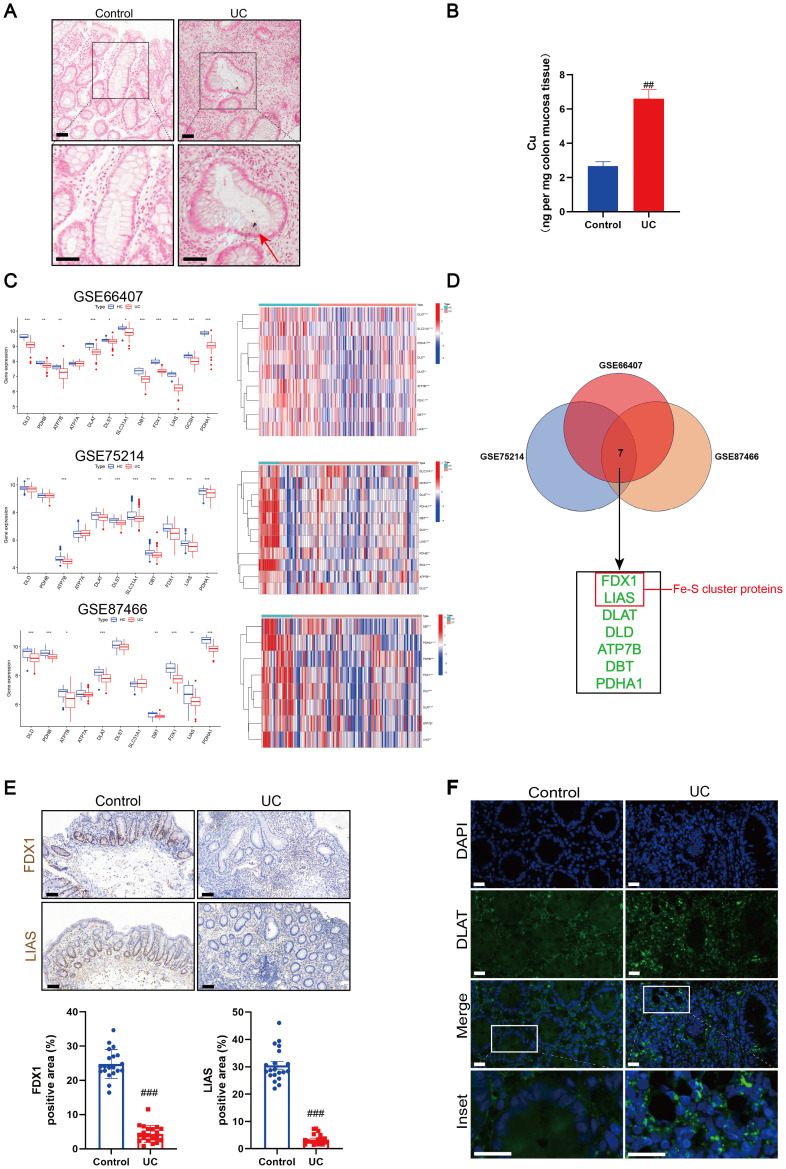
Cuproptosis was induced in ulcerative colitis (UC). **(A)** Rubeanic acid copper staining of intestinal sections in patients with UC and control individuals. The brown section indicated with a red arrow shows copper salt deposition. Scale bar, 50 μm (40 × magnification). **(B)** Copper levels of colonic biopsy tissue were determined by Inductively coupled plasma-mass spectrometry (ICP-MS). **(C)** Heatmap and boxplot of cuproptosis-related genes between UC patients and control individuals in colonic biopsy samples from the dataset. (*) P < 0.05 versus the healthy individuals; (**) P < 0.01 versus the healthy individuals; (***) P < 0.001 versus the healthy individuals. **(D)** Venn diagram of cuproptosis-related differentially expressed genes across three datasets. **(E)** Representative images of immunohistochemical (IHC) staining of ferredoxin 1 (FDX1) and lipoyl synthase (LIAS) in colon tissues from UC patients and control individuals. Scale bar, 50μm (40× magnification). **(F)** Immunofluorescent staining for dihydrolipoamide S-acetyltransferase (DLAT) were performed in the colonic sections of control and UC patients (DLAT - green, DAPI - blue). Scale bar, 20 μm (90 × magnification). Data were shown as mean ± SEM. (##) P < 0.01 versus the control group; (###) P < 0.001 versus the control group.

Recent research has demonstrated the role of copper in immune regulation, such as elevated copper levels in inflamed tissues and abnormal copper metabolism in patients with UC (31–33). As the abnormal accumulation of copper is a key step leading to the occurrence of cuproptosis, we assessed the copper content of the colonic mucosal tissue in patients with UC and healthy individuals. Rubeanic acid-copper staining of colon tissue sections revealed brown copper salt deposits in the colonic crypts of patients with UC (**Figure 1A**). The total copper content in the colonic mucosa was assessed using ICP-MS. Compared to the control group, the copper content in the colonic mucosa of patients with UC was significantly higher (**Figure 1B**). We hypothesized that mucosal cuproptosis was associated with UC. To validate this hypothesis, we searched three datasets (GSE66407, GSE75214, and GSE87466) from the GEO database to compare the transcription of carcinogenesis-related genes between healthy individuals and patients with UC. Genes associated with cuproptosis, such as FDX1, lipoyltransferase 1, LIAS, dihydrolipoamide dehydrogenase (DLD), dihydrolipoamide branched chain transacylase E2 (DBT), glycine cleavage system protein H, dihydrolipoamide S-succinyltransferase (DLST), pyruvate dehydrogenase E1 subunit alpha 1 (PDHA1), DLAT, copper importer SLC31A1, and copper exporters ATP7A and ATP7B, have been reported in earlier research (17). The results revealed that the above cuproptosis-related genes had greatly altered transcript levels in patients with UC compared to those in healthy individuals, suggesting that cuproptosis is associated with UC (**Figure 1C**). Subsequently, a cross-analysis of the significantly altered cuproptosis-related genes across the three datasets yielded seven genes: FDX1, LIAS, DLAT, DLD, ATP7B, DBT, and PDHA1 (**Figure 1D**). Among these, the Fe-S cluster-containing proteins, FDX1 and LIAS, are key regulatory factors in cuproptosis. Immunohistochemistry revealed that the expression of FDX1 and LIAS was lower in the colonic mucosal tissues of UC patients than in healthy individuals (**Figure 1E**). During cuproptosis, excess copper directly binds to acetylated proteins, leading to the oligomerization of DLAT. We observed the presence of DLAT foci in the colons of patients with UC using immunofluorescence, implying the presence of abnormal protein oligomerization (**Figure 1F**).

Based on the above results in patients with UC, we stimulated mice with 2.5% DSS for 1 week to induce experimental colitis. We then detected the copper content in the colonic mucosa of mice. Rubeanic acid-copper staining of mouse colon tissue sections revealed that brown copper salt deposition was positively expressed in the colons of DSS-treated mice (**Supplementary Figure S1A**). The total copper content in the colonic mucosa was measured using ICP-MS. The total copper content in the colonic mucosa of DSS-treated mice significantly increased (**Supplementary Figure S1B**). We targeted the key genes involved in cuproptosis and confirmed that the mRNA and protein levels of both FDX1 and LIAS were significantly decreased in the colonic mucosa of DSS-treated mice (**Supplementary Figures S1C, D**). The loss of FDX1 and LIAS during cuproptosis may have decreased the levels of downstream protein lipoylation. Accordingly, lipoic acid-specific antibodies were used to test the lipoylation of DLAT and DLST to determine the level of protein lipoylation in the colonic mucosa of DSS mice. Colonic mucosal protein lipoylation significantly decreased in DSS-treated mice (**Supplementary Figure S1D**). Immunofluorescence analysis revealed the presence of DLAT foci in the colons of DSS-treated mice, suggesting abnormal protein oligomerization (**Supplementary Figure S1E**). We detected lipoylation-dependent oligomerization of DLAT in the colonic mucosa of DSS-treated mice using non-denaturing gel electrophoresis (**Supplementary Figure S1F**). These data suggested that cuproptosis occurred in the colonic mucosa of mice with DSS-induced colitis. Moreover, DSS administration resulted in inflammatory damage to the colon, characterized by significant leukocyte infiltration, and immunofluorescence showed that the intestinal barrier-related proteins ZO-1 and occludin were decreased (**Supplementary Figures S2A, B**).

Based on these findings, we concluded that cuproptosis induced by damage to the intestinal barrier plays a major role in UC development.

### 3.2 Copper depletion ameliorates DSS-induced colitis in mice

**Figure 2:**
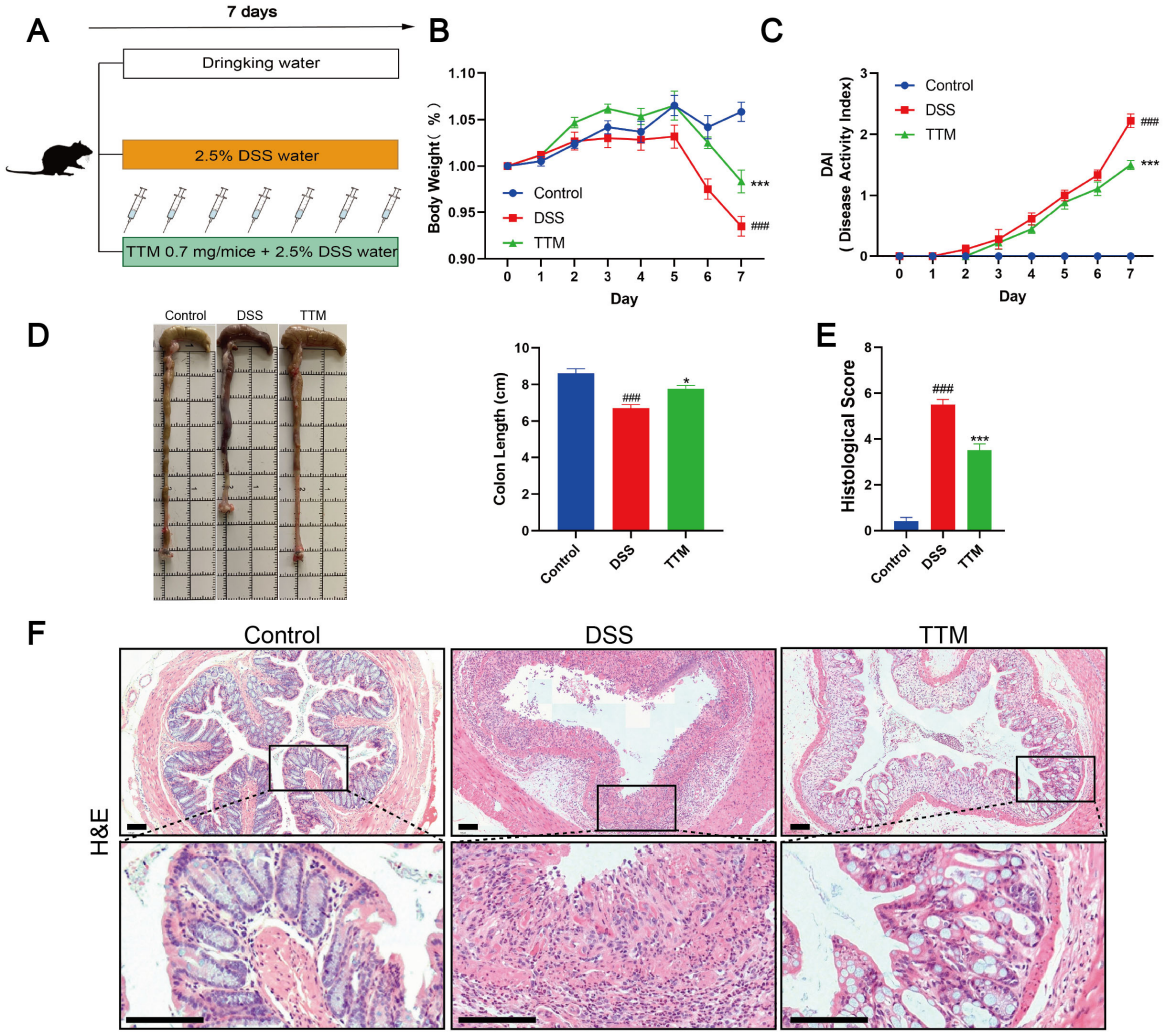
Copper depletion ameliorates dextran sulfate sodium (DSS)-induced colitis in mice. **(A)** Experimental design of the DSS-induced colitis mouse model and the process flowchart for tetrathiomolybdate (TTM) gavage (0.7 mg/mice). Mice were induced with colitis using 2.5% DSS in drinking water for seven consecutive days; control group mice received normal drinking water daily, and TTM was administered via gavage during this period. **(B, C)** Daily changes in body weight and disease activity index (DAI) score from control, DSS, and TTM mice groups. **(D)** Macroscopic observation and length of the colon. **(E, F)** Histological scores and representative H&E images of the colon tissue. Scale bar, 100 μm (15× magnification). Data were shown as mean ± SEM. (###) P < 0.001 versus the control group; (*) P < 0.05 versus the DSS group; (***) P < 0.001 versus the DSS group.

TTM, a copper-depleting agent, was the first cuproptosis inhibitor identified *in vitro* (17). TTM forms inert complexes with copper and albumin in the circulation while enhancing biliary copper excretion (34). To further confirm the causality between cuproptosis and intestinal barrier damage, we treated DSS mice with TTM (**Figure 2A**). We observed that TTM treatment significantly decreased the DAI score and improved weight loss in the experimental colitis mice (**Figures 2B, C**). Moreover, compared to DSS-induced colitis mice, TTM treatment resulted in an increase in colon length (**Figure 2D**) and improved histopathological features, including reduced mucosal ulceration, decreased infiltration of inflammatory cells, damage to crypts, and destruction of the surface epithelium (**Figures 2E, F**). These results indicate that inhibiting cuproptosis by depleting excess copper improves disease outcomes in DSS-induced colitis mice.

### 3.3 Copper depletion ameliorates intestinal barrier damage in DSS-induced colitis mice

**Figure 3:**
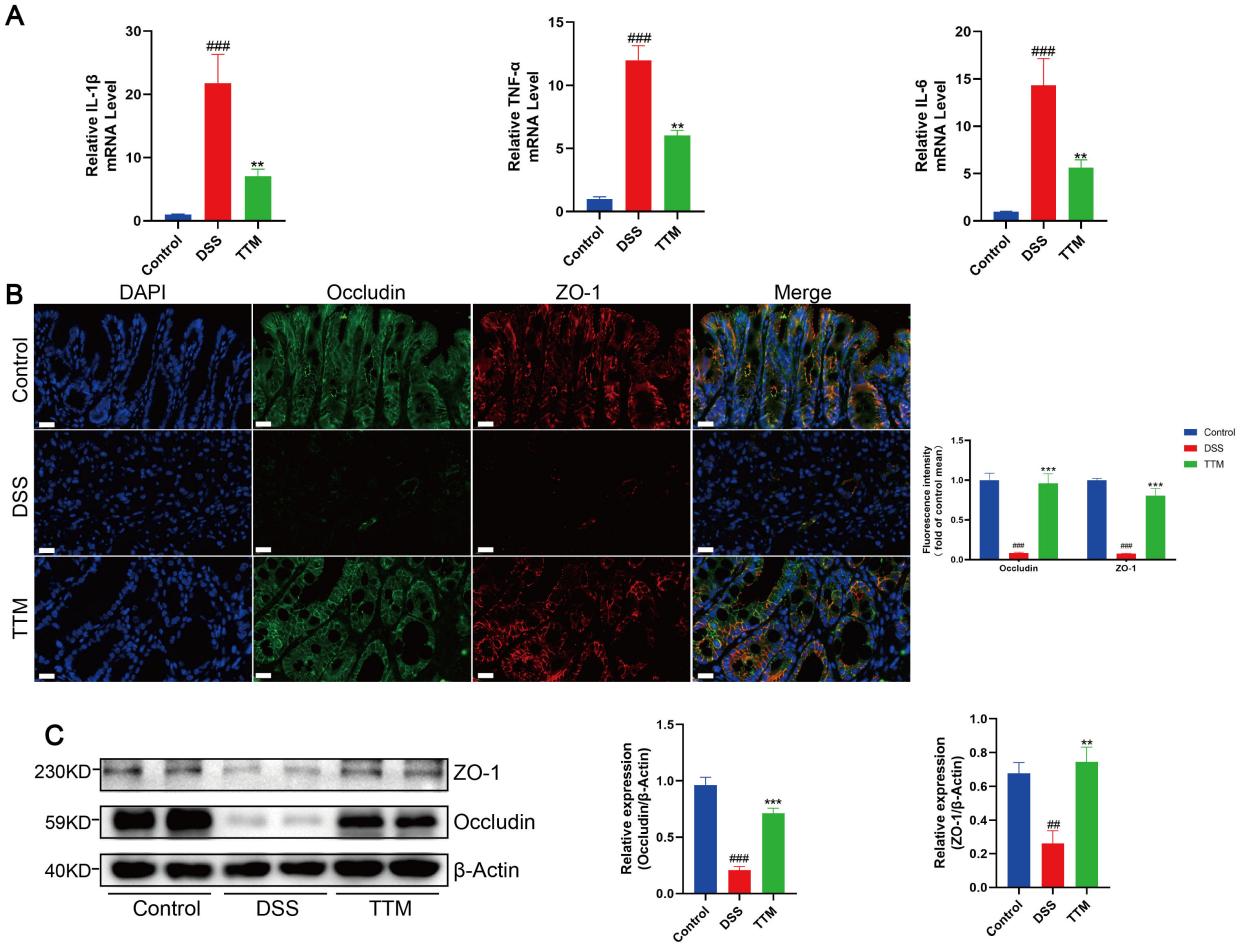
Copper depletion ameliorates intestinal barrier damage in DSS-induced colitis mice. **(A)** mRNA levels of IL-1β, TNF-α and IL-6 were detected by real-time PCR. **(B)** Immunofluorescent staining for zonula occludens 1 (ZO-1); occludin was performed on mouse colonic sections (ZO-1, red; occludin, green; DAPI, blue). Scale bar, 20 μm (90× magnification). **(C)** Western blot analysis of ZO-1 and occludin expression. β-actin was used as the loading control. Data were shown as mean ± SEM. (##) P < 0.01 versus the control group; (###) P < 0.001 versus the control group; (**) P < 0.01 versus the DSS group; (***) P < 0.001 versus the DSS group.

We observed that TTM therapy improved intestinal inflammation in DSS-treated mice, as shown by a major reduction in the levels of inflammatory cytokines IL-6, TNF-α, and IL-1β in the colonic mucosa (**Figure 3A**). Immunofluorescence and western blotting showed that the protein expression levels of the intestinal barrier-associated proteins ZO-1 and occludin were significantly lower in the colonic mucosa of DSS-induced colitis mice than in normal mice (**Figures 3B, C**). TTM treatment markedly increased ZO-1 and occludin protein levels. These results indicated that inhibiting cuproptosis improved intestinal barrier function in DSS-induced colitis mice.

### 3.4 Copper depletion inhibits cuproptosis in the colonic mucosa of DSS-induced colitis mice

**Figure 4:**
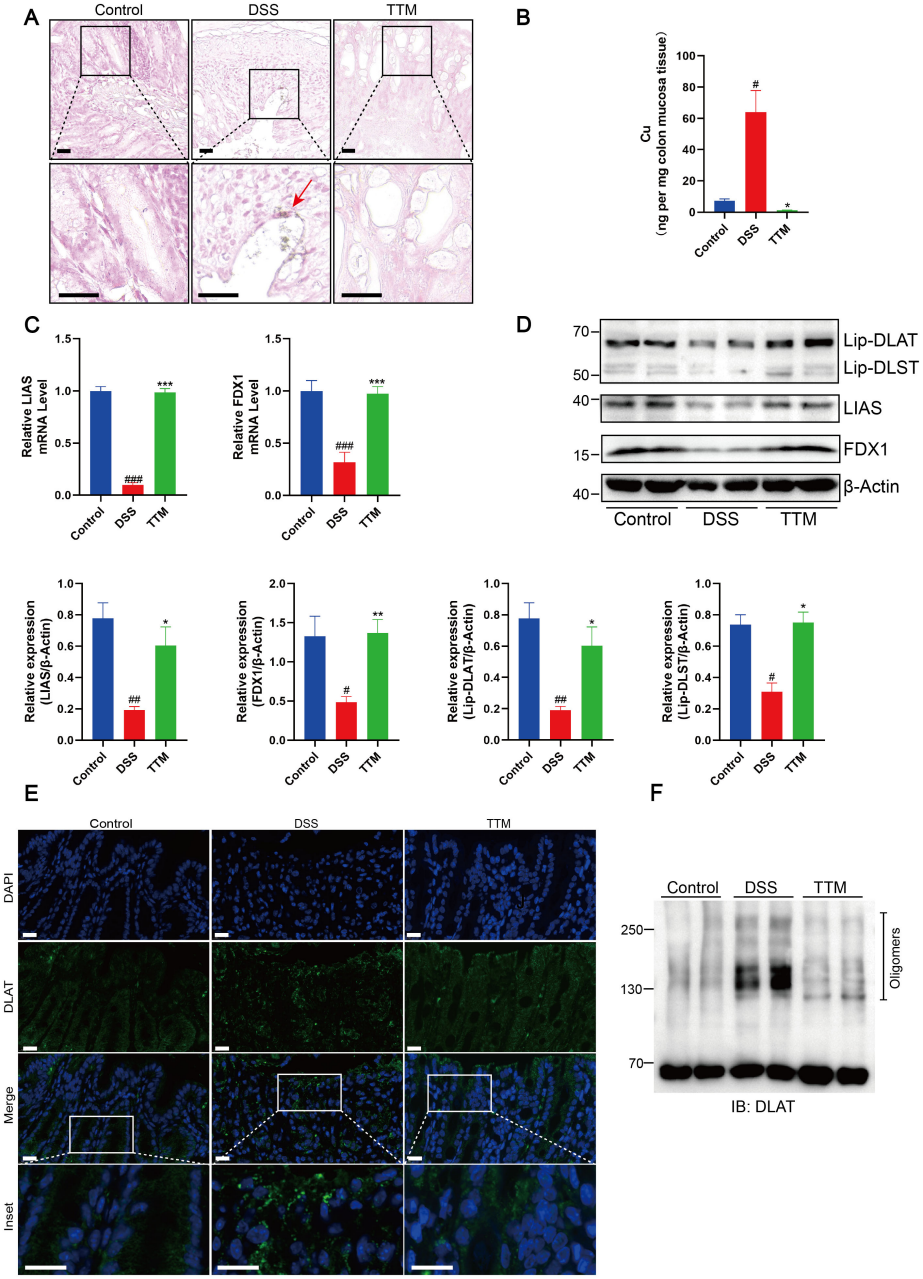
Copper depletion inhibits cuproptosis in colonic mucosa of DSS-induced colitis mice. **(A)** Rubeanic acid-copper staining of intestinal sections from control, DSS-treated, and TTM-treated mice. The brown section indicated with a red arrow represents copper salt deposition. Scale bar, 50 μm (40 × magnification). **(B)** Copper levels in the colonic epithelial tissues of mice were determined using ICP-MS. **(C)** FDX1 and LIAS mRNA levels were detected by real-time PCR. **(D)** Western blot analysis of FDX1, LIAS, and the lipoylated proteins. β-actin was used as the loading control. **(E)** Immunofluorescent staining for DLAT was performed on mouse colonic sections (DLAT, green; DAPI, blue). Scale bar, 20 μm (90× magnification). **(F)** Protein oligomerization in the colonic mucosa of mice was analyzed by immunoblotting. Data were shown as mean ± SEM. (#) P < 0.05 versus the control group; (##) P < 0.01 versus the control group; (###) P < 0.001 versus the control group; (*) P < 0.05 versus the DSS group; (**) P < 0.01 versus the DSS group; (***) P < 0.001 versus the DSS group.

We used copper staining and ICP-MS analysis to confirm that TTM inhibited cuproptosis in the colonic mucosa of mice with colitis by depleting excessive copper accumulation. Following TTM administration, we observed a significant reduction in elevated copper levels in the colonic mucosa of mice with colitis (**Figures 4A, B**). Using qPCR and western blotting, we observed that TTM therapy significantly enhanced the transcriptional and protein expression of FDX1 and LIAS (**Figures 4C, D**). During cuproptosis, the loss of FDX1 and LIAS, which are upstream regulators of protein lipoylation, results in reduced lipoylation levels. TTM administration markedly restored protein lipoylation levels (**Figure 4D**). Moreover, immunofluorescence and non-denaturing gel electrophoresis revealed that TTM administration reduced copper-induced DLAT oligomerization (**Figures 4E, F**).

### 3.5 PA alleviates intestinal inflammation and barrier damage in experimental colitis

**Figure 5:**
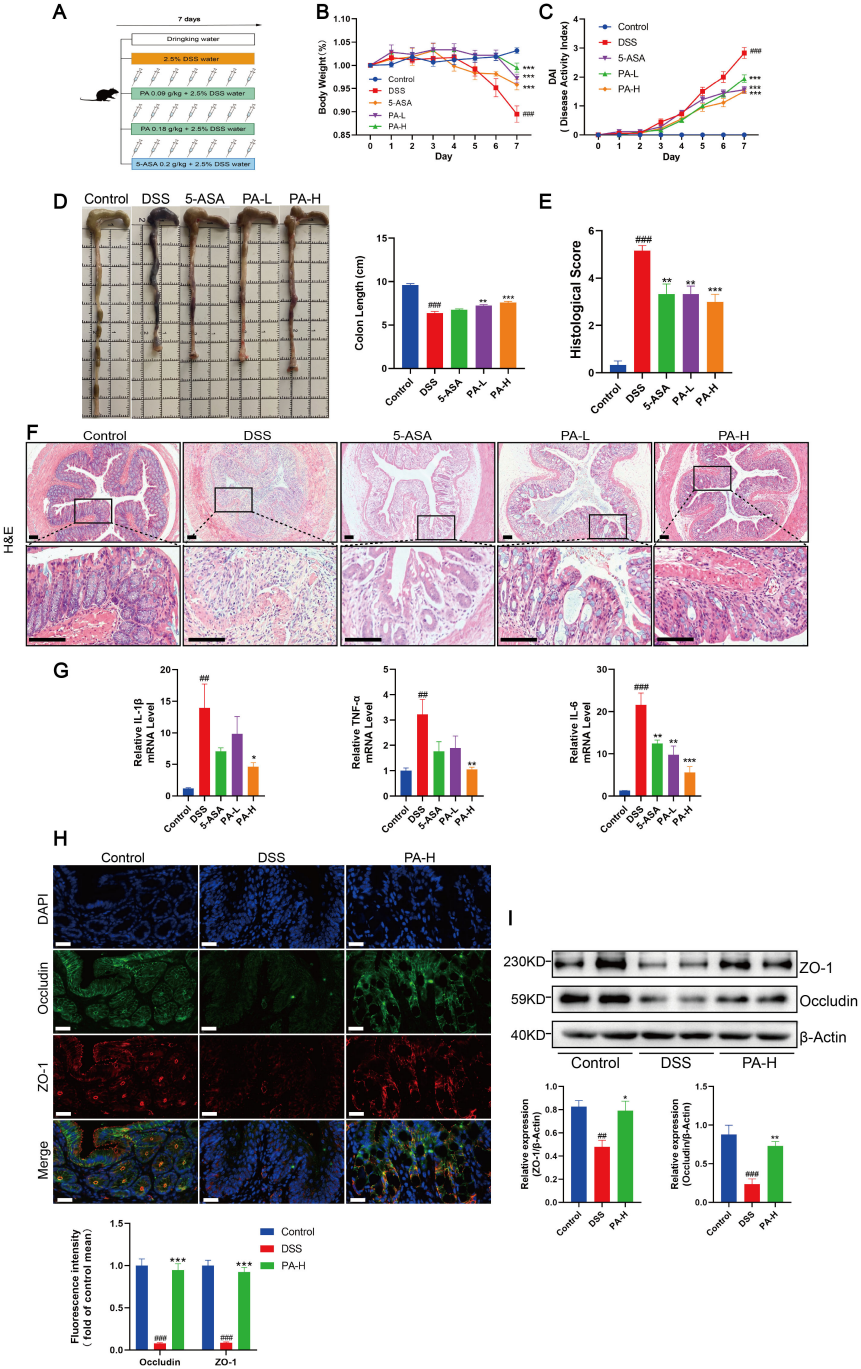
Penicillamine (PA) alleviates intestinal inflammation and barrier damage in experimental colitis. **(A)** Experimental design of the DSS-induced colitis mouse model and process flowchart for PA gavage (0.09 g/kg, 0.18 g/kg) and 5-aminosalicylic acid gavage (5-ASA, 0.2 g/kg). Mice were induced with colitis using 2.5% DSS in drinking water for seven consecutive days, while control group mice received normal drinking water daily. PA was administered via gavage during this period, with 5-ASA serving as a positive control. **(B, C)** Daily changes in body weight and DAI scores for control, DSS, 5-ASA, PA-L, and PA-H mice. D. Macroscopic observation and length of the colon. **(E, F)** Histological scores and representative H&E images of the colon tissue. Scale bar, 100 μm (15× magnification). **(G)** mRNA levels of IL-1β, TNF-α, and IL-6 were detected by real-time PCR. **(H)** Immunofluorescent staining for ZO-1 and occludin were performed in the colonic sections of mice (ZO-1, red; occludin, green; DAPI, blue). Scale bar, 20 μm (90× magnification). **(I)** Western blotting analysis of ZO-1 and occludin. β-actin was used as the loading control. Data were shown as mean ± SEM. (##) P < 0.01 versus the control group; (###) P < 0.001 versus the control group; (*) P < 0.05 versus the DSS group; (**) P < 0.01 versus the DSS group; (***) P < 0.001 versus the DSS group.

We have demonstrated that a copper chelator can inhibit cuproptosis in the colonic mucosa by depleting excess copper, thereby improving intestinal barrier damage in DSS-induced colitis mice. As a chelator of heavy metal ions, PA increases the excretion of various metal ions, including copper. In clinical practice, PA is primarily used to treat copper metabolism disorders and is a key medication for managing conditions such as Wilson’s disease (WD), exerting systemic effects through high-affinity binding to non-ceruloplasmin-bound copper in the plasma (35, 36). To explore whether PA could ameliorate colon inflammation and intestinal barrier damage in mice with DSS-induced colitis, we treated them with low- or high-dose PA (**Figure 5A**). Mice treated with low- or high-dose PA or 5-ASA showed significant improvements in DAI scores and reduced weight loss (**Figures 5B, C**). Compared to DSS-induced colitis mice, administration of PA increased colon length and improved histopathological features, including the reduction of inflammatory cell infiltration, crypt injury, and intestinal epithelial destruction (**Figures 5D–F**). Additionally, high-dose PA treatment significantly reduced the levels of inflammatory cytokines (IL-1β, TNF-α, and IL-6) in the colonic mucosa of the DSS-treated mice (**Figure 5G**) and increased the protein expression levels of the intestinal barrier-related proteins, ZO-1 and occludin (**Figures 5H, I**). These results suggested that PA ameliorated DSS-induced colonic inflammation and barrier damage in mice.

### 3.6 PA reduced cuproptosis in the colonic mucosa of DSS-induced colitis of mice

**Figure 6:**
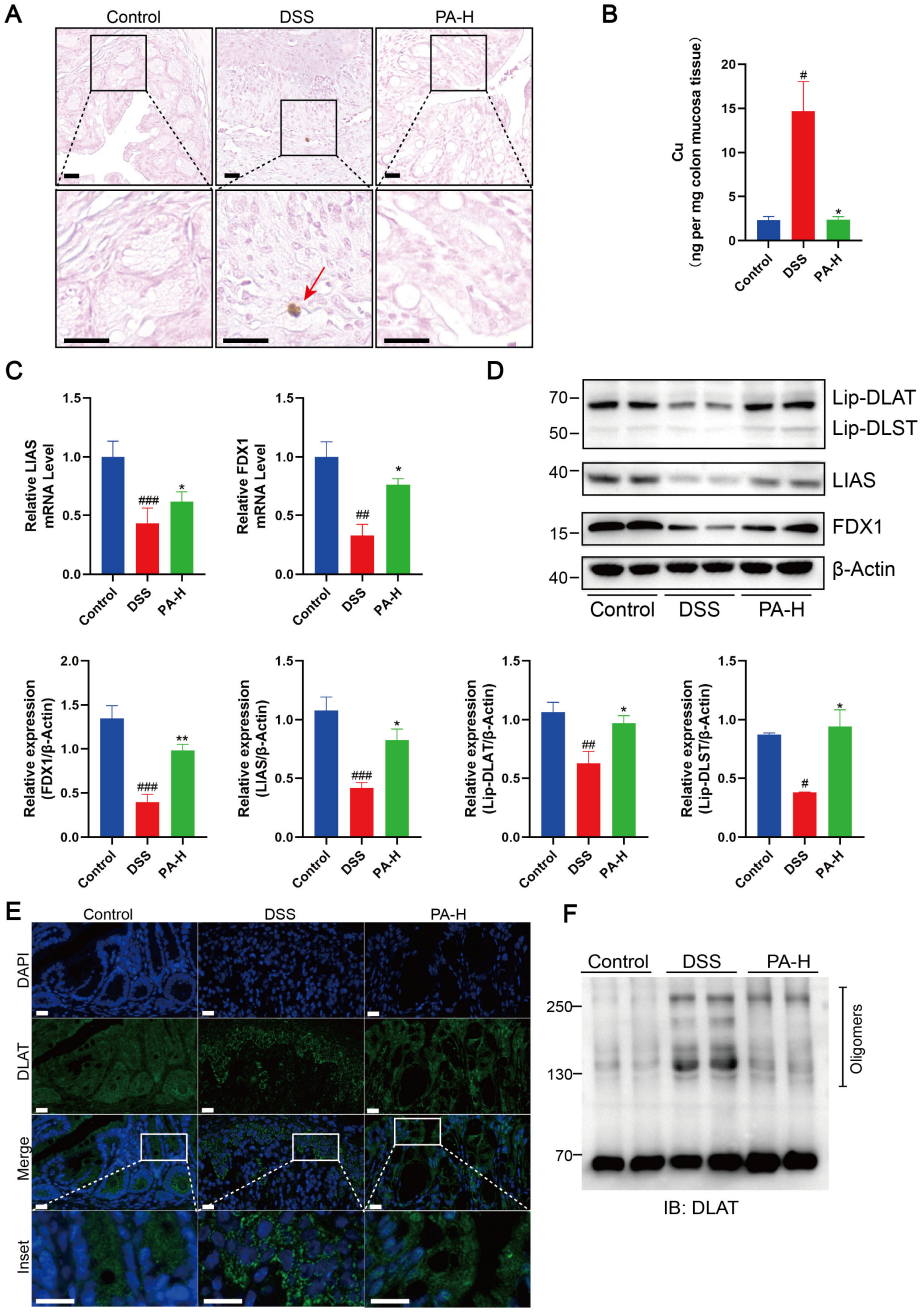
PA reduced cuproptosis in the colonic mucosa of DSS-induced colitis of mice. **(A)** Rubeanic acid-copper staining of intestinal sections from control, DSS-treated, and PA-treated mice. The brown part indicated with a red arrow represents copper salt deposition. Scale bar, 50 μm (40× magnification). **(B)** Copper levels in the colonic epithelial tissues of mice were determined using ICP-MS. **(C)** FDX1 and LIAS mRNA levels were detected by real-time PCR. **(D)** Western blot analysis of FDX1, LIAS, and the lipoylated proteins. β-actin was used as the loading control. **(E)** Immunofluorescent staining for DLAT was performed on mouse colonic sections (DLAT, green; DAPI, blue). Scale bar, 20 μm (90× magnification). **(F)** Protein oligomerization in the colonic mucosa of mice was analyzed by immunoblotting. Data were shown as mean ± SEM. (#) P < 0.05 versus the control group; (##) P < 0.01 versus the control group; (###) P < 0.001, versus the control group; (*) P < 0.05, DSS group; (**) P < 0.01, DSS group.

Rubeanic acid-copper staining of mouse colon tissue sections revealed minimal copper deposition in DSS+PA mice, similar to that in the control group (**Figure 6A**). We measured the total copper content in the colonic mucosa using ICP-MS. Compared to DSS-induced colitis mice, the total copper content in the colonic mucosa of test subjects treated with PA was significantly reduced (**Figure 6B**). Compared with DSS-induced colitis mice, PA treatment significantly reduced the loss of FDX1 and LIAS and significantly increased the level of protein lipoylation (**Figures 6C, D**). Subsequently, using immunofluorescence and non-denaturing gel electrophoresis, we observed that PA treatment reduced copper-induced DLAT oligomerization (**Figures 6E, F**). These results show that PA inhibits cuproptosis in the colonic mucosa of DSS-treated mice.

## 4 Discussion

UC is a chronic inflammatory disease characterized by the disruption of the intestinal barrier, which initially manifests as intestinal epithelial dysfunction (37, 38). Among the various processes involved in the pathogenesis of UC, cell death is significant (39–42). Cuproptosis is a newly identified type of regulated cell death resulting from an overload of copper (16). Excessive intracellular copper triggers the aggregation of lipoylated DLAT (a key component of the mitochondrial TCA cycle), induces proteotoxic stress, and culminates in a novel cell death pathway termed cuproptosis. The hallmark of cuproptosis is aberrant copper accumulation, which ultimately leads to cell death. However, whether the characteristic ultrastructural alterations in subcellular organelles manifest during cuproptosis remains unclear. Previous studies have only predicted the correlation between cuproptosis and UC using data analysis (23, 43). Therefore, there is an urgent need to elucidate the causality between cuproptosis and intestinal barrier damage in UC while investigating novel therapeutic approaches for UC treatment.

Copper accumulation is necessary for triggering cuproptosis. Previous studies have reported abnormal copper accumulation in individuals with UC compared with that in normal individuals (22). Based on copper staining and ICP-MS analysis, our results confirmed that higher levels of copper were observed in the colonic mucosa of patients with UC and mice with DSS-induced colitis. To further investigate the possible targets of cuproptosis in UC, three GEO datasets were analyzed for cuproptosis-related genes. Seven overlapping genes, including FDX1 and LIAS, were differentially expressed in patients with UC. During cuproptosis, excess copper leads to the loss of Fe-S cluster-containing proteins FDX1 and LIAS, resulting in the oligomerization of DLAT, in a process potentially linked to hallmark immune dysregulation of UC (16). This connection is supported by Huang et al., who demonstrated that FDX1 and LIAS are negatively correlated with immune infiltration in UC, suggesting that their suppression may exacerbate inflammation (23). In this study, we observed that the transcription and protein expression of FDX1 and LIAS were significantly decreased in the colonic mucosa of patients with active UC with Mayo endoscopic scores ≥2 and in mice with DSS-induced colitis. This preliminary observation indicates a complex relationship between cuproptosis-related proteins and disease severity. Future studies with larger cohorts should clarify these links through correlation analyses between cuproptosis-associated genes or proteins and clinical severity indices.

Cuproptosis represents a regulated cell death pathway that is intrinsically linked to mitochondrial metabolism, a relationship that was further illuminated by our findings. Prior mechanistic studies using ATP7B-knockout models have demonstrated that copper accumulation induces a significant suppression of DLAT, an enzyme essential for mitochondrial respiration (17). Our transcriptomic analysis of three independent UC datasets consistently revealed downregulated DLAT expression in patient cohorts, indicating that copper overload in UC directly impairs mitochondrial protein homeostasis. This copper-mediated DLAT dysregulation provides a molecular bridge between the observed cuproptosis elevation in DSS models and the established mitochondrial dysfunction in UC pathogenesis. Concurrently, we observed increased DLAT oligomerization in these tissues. Our results revealed reduced lipoylation of DLAT and DLST in mice with DSS-induced colitis. Studies carried out previously have established that FDX1 and LIAS positively regulate the lipoylation of DLAT and DLST (17). Oligomerization of DLAT is dependent on lipoylation, which results in the formation of a tight bond with copper. In DSS-induced colitis mice, the level of DLAT lipoylation was significantly reduced, but excess copper directly bound to and induced the oligomerization of lipoylated DLAT. These results suggest that the aberrant accumulation of copper, which triggers cuproptosis, significantly contributes to the pathogenesis of UC.

Subsequently, TTM-induced copper depletion was performed to confirm the causality between copper depletion and UC. We observed that TTM significantly improved weight, DAI score, colon length, and pathological symptoms in mice. TTM significantly reduced colonic inflammation and the levels of IL-6, IL-1β, and TNF-α in colitis mice. Our results demonstrated that copper depletion inhibited cuproptosis in the DSS-induced colitis mice, as indicated by a major reduction in copper content and DLAT oligomerization, restoration of FDX1 and LIAS levels, and an increase in corresponding protein lipoylation levels in the colonic mucosa after TTM treatment. In addition, we observed that TTM rescued the loss of intestinal barrier-associated proteins ZO-1 and occludin. ZO-1 and occludin are major components of tight junctions that form the mechanical barrier in the intestine (44–46). In UC, the tight junctions of the intestinal barrier are disrupted, resulting in the penetration of pro-inflammatory substances from the intestinal lumen. This activates the mucosal immune response, leading to inflammation and tissue damage (47). These results indicate that cuproptosis plays a crucial role in the pathogenesis of UC and suggest a new therapeutic approach for UC. The inhibition of cuproptosis by depleting excess copper appears to be a feasible strategy for improving intestinal barrier disruption.

PA was the first oral drug used to treat WD. PA can chelate copper and is widely used in clinical practice due to its affordability and marked efficacy (48). It binds to copper ions in the body to form soluble complexes that enhance copper excretion and reduce its accumulation (49). Therefore, it is an effective treatment for copper metabolism disorders (50). Our study showed that PA improved colitis in mice, as evidenced by improvements in disease-related indicators, including weight loss, DAI scores, pathological manifestations, and levels of pro-inflammatory mediators (IL-1β, IL-6, TNF-α). We demonstrated that PA effectively inhibited cuproptosis in the colonic mucosa of colitis mice, as shown by the reduction in abnormally elevated copper levels in the colonic mucosa, restoration of FDX1, LIAS, and protein lipoylation levels, and decreased DLAT oligomerization. Moreover, PA improved intestinal barrier damage in mice with DSS-induced experimental colitis by increasing the expression levels of ZO-1 and occludin. Although our results indicate that PA prevents cuproptosis in DSS-induced colitis mice, thereby reducing intestinal barrier injuries, its documented anti-inflammatory effects may synergistically enhance mucosal healing (51). Further studies are needed to explore the potential anti-inflammatory and antioxidant effects of PA on UC.

Although this study established cuproptosis as a pathogenic mechanism of UC, several limitations must be considered. Although both UC and Crohn’s disease are forms of inflammatory bowel disease, we exclusively investigated the relationship between UC and cuproptosis. This focus was determined by the core pathological mechanism of UC, namely the disruption of the intestinal epithelial barrier and the critical role of cell death modalities in driving this barrier impairment, which aligns mechanistically with the pathogenesis of cuproptosis. Future studies should explore the analogous mechanisms in Crohn’s disease using appropriate model systems when sufficient clinical specimens are available. The clinical sample size limited our ability to determine which cuproptosis-related proteins exhibit the strongest correlation with disease severity, potentially affecting the clinical translatability of these biomarkers. Furthermore, while the DSS-induced colitis model is well validated for acute inflammation studies, its exclusive application may not fully replicate the chronic immune dysregulation and epithelial remodeling observed in human UC. Future investigations should prioritize expanded clinical cohorts for severity correlation analyses alongside complementary *in vitro* models using intestinal epithelial-specific cuproptosis induction, thereby strengthening the mechanistic bridge between experimental findings and human pathophysiology.

## 5 Conclusion

**Figure 7:**
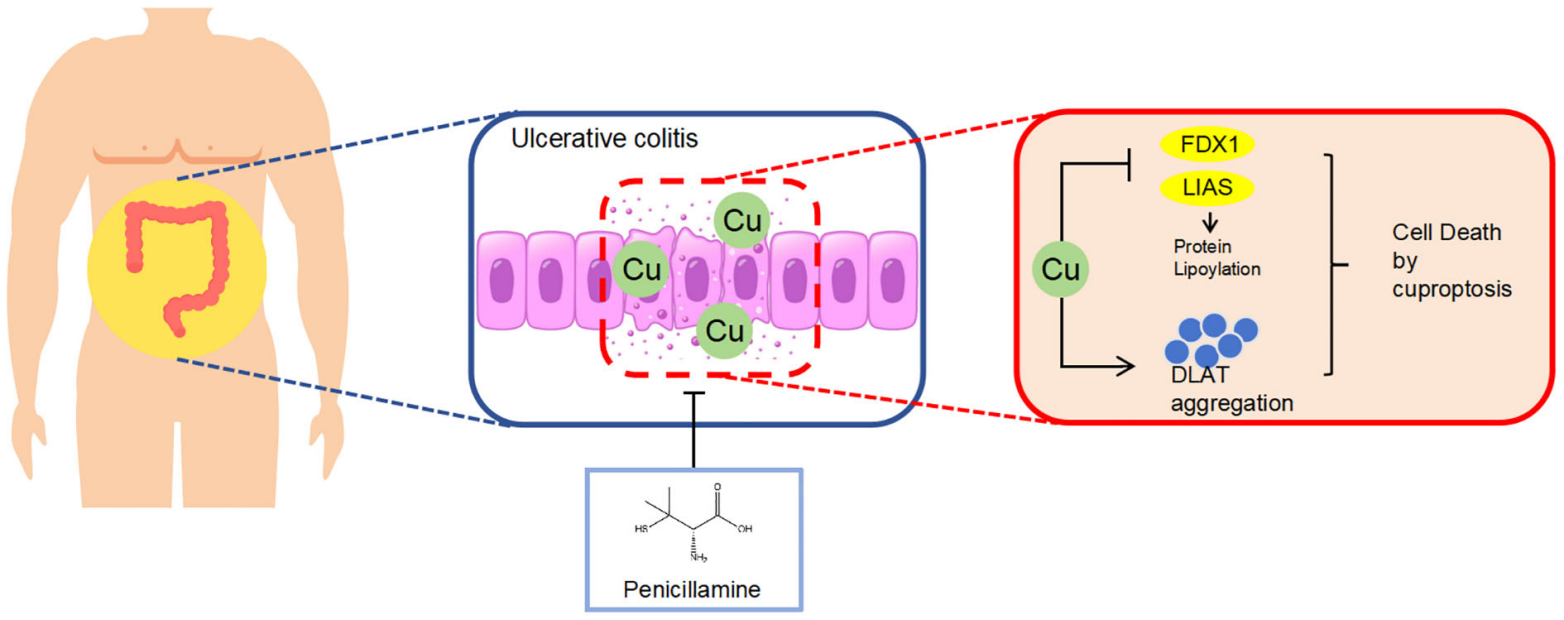
Schematic diagram illustrating the molecular mechanism by which PA ameliorates DSS-induced colitis by inhibiting cuproptosis in the colonic mucosa through the depletion of excess copper.

Our study is the first to demonstrate the accumulation of copper in the colonic mucosa, resulting in the loss of Fe-S cluster-containing proteins FDX1 and LIAS and a reduction in DLAT oligomerization leading to cuproptosis in DSS-induced colitis mice. PA inhibited cuproptosis in DSS-induced colitis mice, thereby lowering intestinal barrier disruption (**Figure 7**). Our results provide preclinical evidence for expanding the clinical application of PA and offer a new approach for treating UC.

## Data Availability

The datasets presented in this study can be found in online repositories. The names of the repository/repositories and accession number(s) can be found in the article/**Supplementary Material**.
