# Supplementary material for: Penicillamine ameliorates intestinal barrier damage in dextran sulfate sodium-induced experimental colitis mice by inhibiting cuproptosis
Source: Front Immunol. 2025 Sep 3;16:1580963. doi: 10.3389/fimmu.2025.1580963 (PMC12440932; doi:10.3389/fimmu.2025.1580963)
Supplement: Supplementary file 1 [file Table1.docx]

Supplementary Materials 1

1. **Antibodies reagents**

FDX1 (abcam ab108257), Lipoic acid (abcam ab58724), LIAS (Proteintech 11577-1-AP), DLAT (Proteintech 13426-1-AP), β-Actin (Proteintech 66009-1-lg), Occludin (Proteintech 80545-1-RR), ZO-1 (Proteintech 21773-1-AP)

1. **The detailed scoring criteria for DAI**

| **Parameter** | **Score 0** | **Score 1** | **Score 2** | **Score 3** | **Score 4** |
| --- | --- | --- | --- | --- | --- |
| **Weight Loss (%)** | **<1%** | **1–5%** | **5–10%** | **10–15%** | **>15%** |
| **Stool Consistency** | **Normal, formed** | **Soft but formed** | **Loose (mild diarrhea)** | **Watery (moderate diarrhea)** | **Liquid (severe diarrhea)** |
| **Rectal Bleeding** | **No blood (Hemoccult −)** | **Trace blood (Hemoccult +)** | **Visible blood in stool** | **Moderate bleeding** | **Severe bleeding (frank blood)** |

**DAI = (Weight loss + Stool consistency + Rectal bleeding) / 3**

1. **Primer sequences**

FDX1_F：TGGAGGTGAGACTAATACCTT

FDX1_R：TTGAGAGAGCATTTGAGGT

LIAS_F：ACACTCCTGAGACCTAAAAG

LIAS_R：CATGTACTCTTGTCCGGG

IL-1β_F：GAAATGCCACCTTTTGACAGTG

IL-1β_R：TGGATGCTCTCATCAGGACAG

TNF-α_F：CCTGTAGCCCACGTCGTAG

TNF-α_R：GGGAGTAGACAAGGTACAACCC

IL-6_F：CTGCAAGAGACTTCCATCCAG

IL-6_R：AGTGGTATAGACAGGTCTGTTGG

β-Actin _F：GGCTGTATTCCCCTCCATCG

β-Actin _R：CCAGTTGGTAACAATGCCATGT

**4. Reaction Volume**

| **reagent** | **Volume** |
| --- | --- |
| **2×Hieff® qPCR SYBR Green Master Mix (Low Rox Plus)** | **5μl** |
| **Forward primer** | **0.2μl** |
| **Reverse primer** | **0.2μl** |
| **RNAase-free dd_H2O_** | **3.6μl** |
| **cDNA** | **1μl** |

**Thermal Profile**

- Initial Denaturation: 95°C for 30 sec

- Amplification (40 cycles):

95°C for 10 sec → 60°C for 30 sec

- Melt Curve:

95°C for 15 sec → 60°C for 60 sec → 95°C for 15 sec

**5. Inductively coupled plasma-Mass Spectrometry**

Copper quantification was performed using an Agilent 7900 ICP-MS with the following parameters:RF power: 1550 W; Plasma gas: 15 L/min; Carrier gas: 0.99 L/min; Sampling depth: 8 mm; Acquisition mode: Spectrum (3 points/peak); Dwell time: 0.1 s; Replicates: 3. Germanium-72 (⁷²Ge) was used as the internal standard at 20 μg/L to correct for matrix effects and instrumental drift. Calibration standards (0-200 μg/L Cu-63) were prepared in 2% HNO₃ matrix-matched to samples. All samples analyzed in triplicate (n=3 technical replicates). Relative Standard Deviation (RSD) < 5% for all replicates. Mean Cu concentrations from triplicate measurements were used for statistical analysis. Between-group differences were assessed by one-way ANOVA. The method detection limit (MDL) was 0.08 μg/L. No samples fell below MDL in this study. Had values been undetectable, they would have been reported as <MDL and excluded from statistical comparisons.

1. **Isolation of colonic epithelial cells**

The whole colon of mice was cut into 2-3 mm lengths and subjected to 10ml digestive solution (300U/ml collagenase Ⅺ (Merck), 0.1mg/ml dispase (Merck), in HBSS), followed shaking vigorously at 37℃ for 30 minutes. Next, tissue/ digestive solution was centrifuged at 1200 revs/min for 3 minutes and the supernatant was discarded. The precipitate was resuspended with 10ml dispersive solution (5% fetal bovine serum, 2% sorbitol, in DMEM medium) and vigorously pipetted 150 times and let rest for 1minute, then removed the supernatant carefully to a new 50ml tube, added new dispersive solution into the precipitates, repeated this whole step three times. Centrifuged the collected supernatant at 300 revs/min for 3 minutes, discarded the supernatant and kept the precipitates for further utilization.
