# Supplementary material for: Penicillamine ameliorates intestinal barrier damage in dextran sulfate sodium-induced experimental colitis mice by inhibiting cuproptosis
Source: Front Immunol. 2025 Sep 3;16:1580963. doi: 10.3389/fimmu.2025.1580963 (PMC12440932; doi:10.3389/fimmu.2025.1580963)
Supplement: Supplementary file 2 [file Table2.docx]

Supplementary Materials 2

**
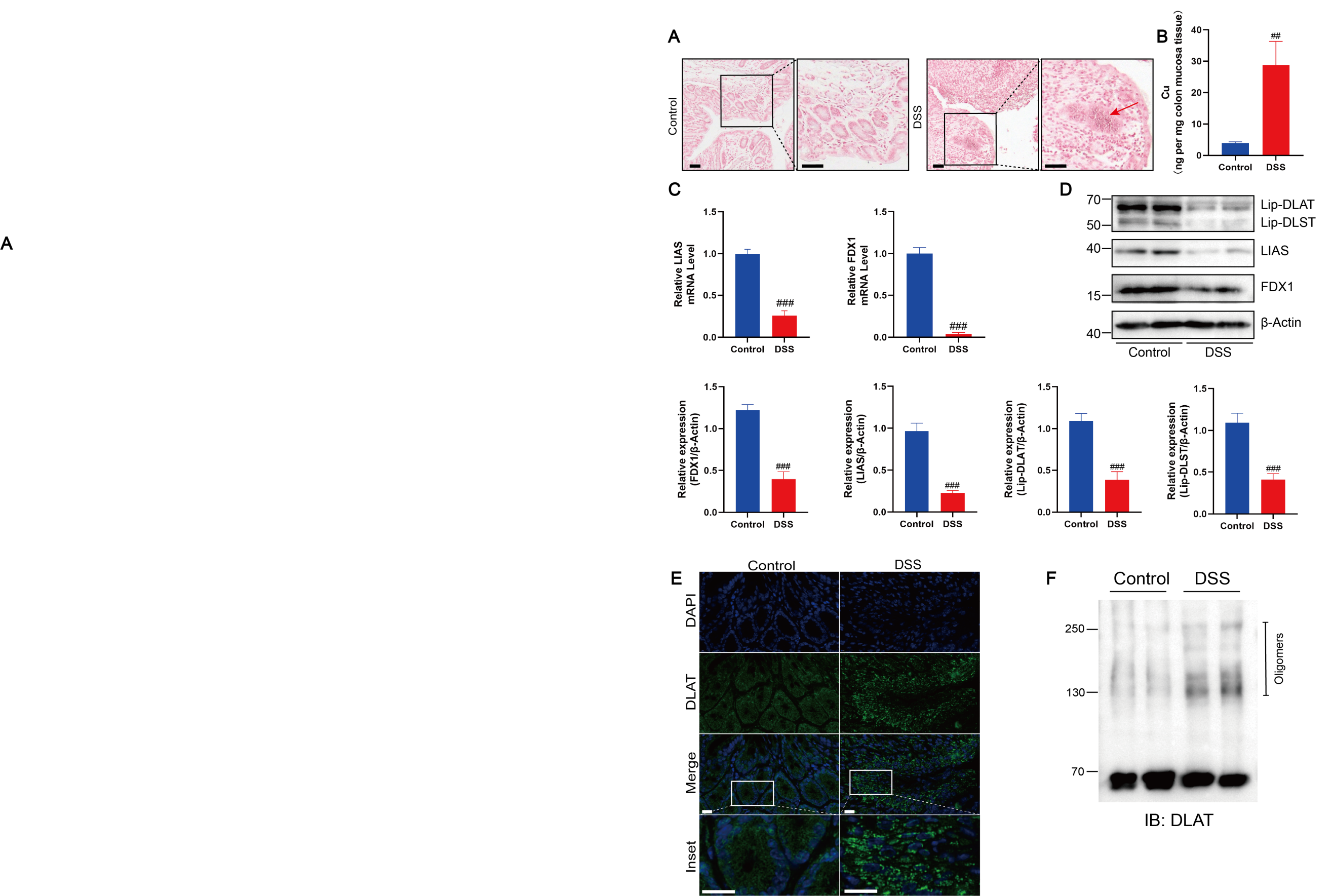
**

**Figure S1. Cuproptosis was induced in DSS-induced colitis of mice.** (A) Rubeanic acid copper staining of intestinal sections with control and dss-treated. The brown part is the copper salt deposition. Scale bar, 50μm (40× magnification). (B) Copper levels of colonic epithelial tissues of mice were determined by LCP-MS. (C) mRNA levels of FDX1 and LIAS were detected by real-time PCR. (D) Western blotting analysis of FDX1, LIAS and lipoylated proteins. β-actin was used as the loading control. (E) immunofluorescent staining for DLAT were performed in the colonic sections of mice (DLAT - green, DAPI - blue). Scale bar, 20μm (90× magnification). (F) protein oligomerization in the colon mucosa of mice was analyzed by immunoblotting. Data were shown as mean ±SEM. (##) P < 0.01 versus the control group; (###) P < 0.001 versus the control group.

**
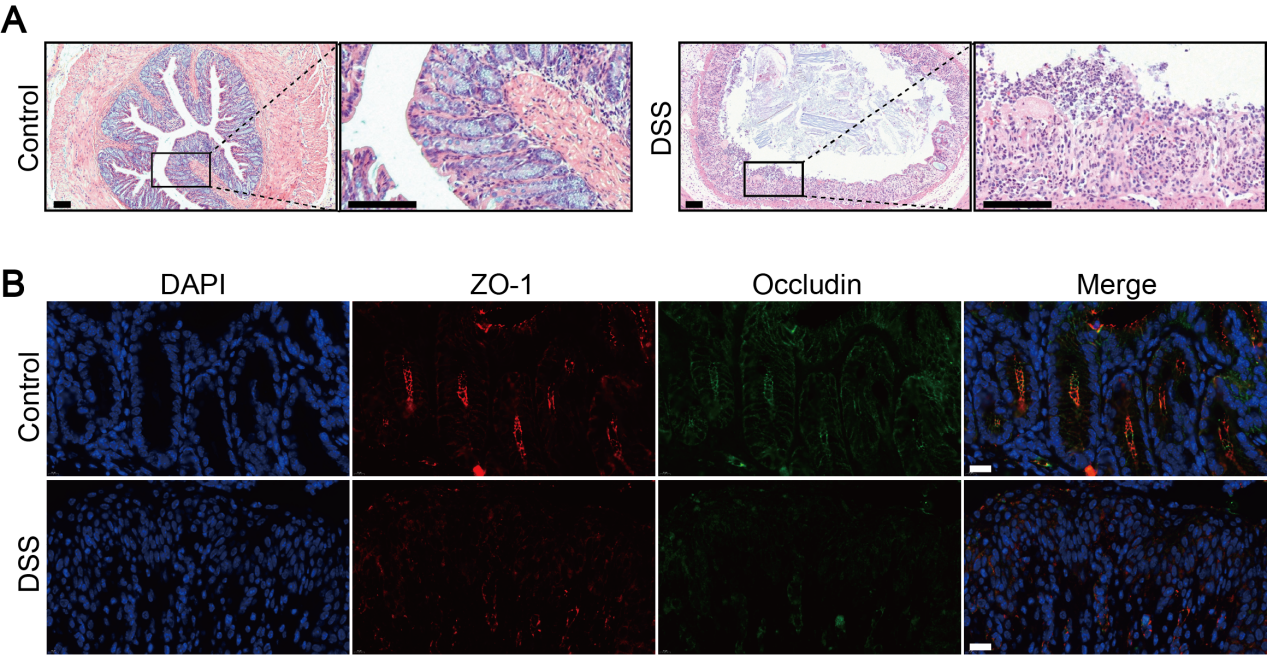
Figure S2. Experimental colitis in mice induced by DSS.** (A) Representative H&E images of the colon tissue. Scale bar, 100μm (15× magnification). (B) Immunofluorescent staining for ZO-1 and Occludin were performed in the colonic sections of mice (ZO-1 - red, Occludin - green, DAPI - blue). Scale bar, 20μm (90× magnification).


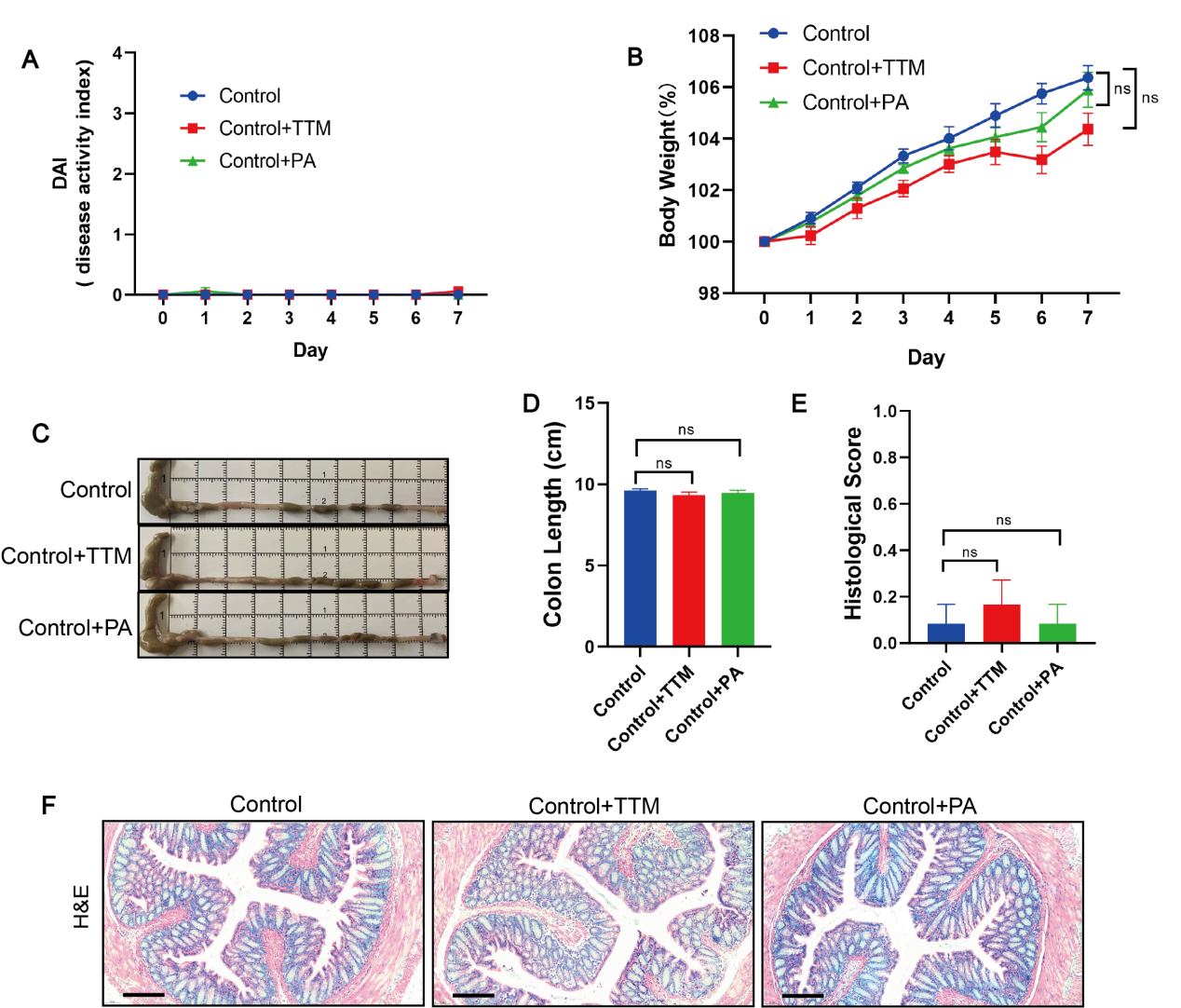
**Figure S3. No physiological impact of TTM/PA monotherapy in healthy mice.** (A, B) Daily changes in body weight and DAI score from different groups of mice. (C) Macroscopic observation and length of the colon. (E, F) Histological scores and Representative H&E images of the colon tissue. Scale bar, 100μm (15× magnification). Data were shown as mean ±SEM. (ns) P ＞0.05 versus the control group.

**Supplementary Table 1. Clinical patient characteristics.**

|  | Healthy Volunteers | UC |
| --- | --- | --- |
| Number | 20 | 20 |
| Gender (m/f) | 10/10 | 11/9 |
| Age (year) | 30 (18–47) | 39 (27–65) |
| Mayo endoscopic score | - | 2 (2–3)^*^ |

Values are reported as mean and (range); ^*^Only 6 UC patients who completed colonoscopy completed the Mayo endoscopic score.
